# Analysis of Smile Aesthetic Changes With Fixed Orthodontic Treatment

**DOI:** 10.7759/cureus.32612

**Published:** 2022-12-16

**Authors:** Ruchi Saini, Neha Thakur, Reshu Jindal Goyal, Kriti Sharma Rai, Hiroj Bagde, Ashwini Dhopte

**Affiliations:** 1 Department of Orthodontics and Dentofacial Orthopedics, Institute of Dental Studies and Technologies (IDST) Dental College, Ghaziabad, IND; 2 Department of Orthodontics and Dentofacial Orthopedics, Kalka Dental College, Meerut, IND; 3 Department of Orthodontics, Private Practice, Delhi, IND; 4 Department of Periodontology, Rama Dental College and Research Centre, Kanpur, IND; 5 Department of Oral Medicine and Radiology, Rama Dental College and Research Centre, Kanpur, IND

**Keywords:** laymen, general dentists, orthodontists, fixed orthodontic treatment, smile

## Abstract

Introduction: The mouth and teeth are vital in facial aesthetics and the face as a whole is the most critical individual component determining one's physical look. As dentists, we need to be aware that this might significantly alter the care given to a patient since the patient's and the dentist's ideas of beauty may be quite different. This study sought to ascertain how the general public, general dental specialists, and orthodontics all rated the attractiveness of patients who had received orthodontic treatment using a visual scale, as well as how the facial reference could be used to gauge the improvement in smiles.

Methodology: The attractiveness of 80 continuously treated patients was assessed by comparing their pre- and post-treatment posed grin images. The attractiveness of participants' smiles was evaluated using the Visual Simple Scale, which measures affect, and the Graph of Facial Stylish Reference, which measures objectively. “Making a Jazzy Face Chart" - The analysts were able to fairly evaluate changes in elements such as the symmetry of the smile twist, the position of the gingival peak of the front teeth relative to each other, the height of the incisal edges of the front teeth, the width of the connector band of the front six teeth, and the general tip of the front teeth by referring to photographs taken during treatment. We put the Visual Basic Scale to the test by having five male orthodontists, five male general dental specialists, and five male laypeople rate how much of an improvement they noticed between the before and after photos of a patient's smile.

Results: According to the findings of the first section of the research, orthodontic treatment generally leads to an increase in all indicators. Some instances, however, demonstrated worsening in characteristics such as grin arc consonance, gingival zenith location relative to each other, and incisal edge height. The second half of this research revealed that the opinions of orthodontic specialists, general dentists, and the general public varied with regard to the beauty of a smile.

Conclusion: Based on this research, we can say the following: Orthodontic treatment led to improvements in many of the variables that contribute to smile attractiveness, factors include the interproximal width of contact area, the height of the incisal margins of the front teeth, and the location of the gingival zenith in relation to one another, to name a few. The results of this research support the idea that the aesthetics of the smile should be assessed at the last stages of orthodontic treatment when fine adjustments are being made.

## Introduction

The mouth and teeth are regarded as crucial in facial aesthetics [1,2] and the face as a whole is the most significant individual component defining one's physical appearance. It is crucial to improve the cosmetic results of orthodontic treatment, and this may be done by understanding the factors that contribute to the harmony between teeth and soft tissues during a beautiful smile [3]. As dentists, we need to be aware that this might significantly alter the care given to a patient since the patient's and the dentist's ideas of beauty may be quite different. One of the first and most effective methods of boosting a person's confidence and happiness via their outward appearance. We all agree that a winning grin must be lively, energetic, and youthful-looking [3]. The lips curl inward to create a grin. A smile is made up of several different parts, including the incisal margins, the gingival embrasures, the gingival height of contour, and the inter-proximal contact zones. A consonant arc, especially one accompanied by a smile, is more attractive than a non-consonant one [4].

The gingival show, gingival relative heights, and gingival shape are additional aspects that contribute to the overall aesthetics of a smile in all age groups globally [4]. Most people who get braces are looking to improve their smile and general facial appearance. In the past decade, analysis of the smile and the creation of new smiles have become integral parts of orthodontic diagnosis and treatment planning [5]. Thanks to recent developments in technology, orthodontists may now include data on the patient's dynamic lip-tooth interaction on their list of issues and in their biomechanical plan. Success rates, and the likelihood of failure, may be increased by the use of simple and trustworthy processes, which can even help to eradicate performance faults. In this regard, the Diagram of Facial Aesthetic References (DFAR) might be a useful supplementary diagnostic tool.

The purpose of this study was for the researchers to determine how orthodontics, general dental specialists, and the general public all rated the attractive quality of patients who underwent orthodontic therapy using a visual scale, as well as how the facial reference can be used to measure the improvement in smiles.

## Materials and methods

The study was carried out in the Department of Orthodontics and Dentofacial Orthopedics, which was approved by the ethical committee of the Institute of Dental Studies and Technologies (IDST) Dental College with reference number 2021/345. We chose two photos at random from the digital collection: one frontal, unposed, pre-treatment photograph, and one post-treatment photo with the subject's head in its natural posture. Inclusion also requires the absence of craniofacial deformities or other diseases and a normal upper lip length (with a balanced face, the length of the upper lip (distance from subnasale to stomion) is equal to one-third of lower facial height (subnasale to menton)).

Ultimately, 80 individuals with a range of malocclusions who were treated in succession were included in the sample. Fifty-two patients out of the total 80 had received extraction treatment (Table 1).

**Table 1:** Improvement in smile aesthetics with the treatment of all patient data

Patient number	Consonance of the smile arc	Position of the gingival zenith of the anterior teeth relative to each other	Relative height of the incisal edges of the anterior teeth	Width of the connector band in the anterior six teeth	Relative tip of the teeth
Patient 1	Improved	Improved	Improved	Improved	Improved
Patient 2	Improved	Worsened	Improved	Improved	Improved
Patient 3	Improved	No change	Improved	Improved	Improved
Patient 4	Worsened	Improved	Worsened	Improved	Improved
Patient 5	Improved	Improved	Improved	Improved	Improved
Patient 6	Improved	Improved	Improved	Improved	Improved
Patient 7	Improved	Improved	Improved	Improved	Improved
Patient 8	No change	No change	No change	Improved	Improved
Patient 9	Improved	Improved	Improved	Improved	Improved
Patient 10	Improved	Improved	Improved	Improved	Improved
Patient 11	Improved	Improved	Improved	Improved	Improved
Patient 12	Improved	No change	Improved	Improved	Improved
Patient 13	Improved	Improved	Improved	Improved	Improved
Patient 14	Improved	Improved	Improved	Improved	No change
Patient 15	Improved	Improved	Improved	Improved	Improved
Patient 16	Improved	Improved	Improved	Improved	Improved
Patient 17	Improved	Improved	Improved	Improved	Improved
Patient 18	Improved	Improved	Improved	Improved	Improved
Patient 19	Improved	Improved	Improved	Improved	Improved
Patient 20	Improved	Improved	Improved	Improved	Improved
Patient 21	Improved	Improved	Improved	Improved	Improved
Patient 22	Improved	Improved	Improved	Improved	Improved
Patient 23	Improved	Improved	Improved	Improved	Improved
Patient 24	Improved	No change	Improved	Improved	Improved
Patient 25	Improved	Improved	Improved	Improved	Improved
Patient 26	Improved	Improved	Improved	Improved	Improved
Patient 27	Improved	Improved	Improved	Improved	Improved
Patient 28	Improved	Improved	Improved	Improved	Improved
Patient 29	Improved	Improved	Improved	Improved	Improved
Patient 30	No change	Improved	Improved	Improved	Improved
Patient 31	Improved	Improved	Improved	Improved	Improved
Patient 32	Improved	Improved	Improved	Improved	Improved
Patient 33	Improved	Improved	Improved	Improved	Improved
Patient 34	Improved	Worsened	Improved	Improved	Improved
Patient 35	Improved	Improved	Improved	Improved	Improved
Patient 36	Improved	Improved	Improved	Improved	Improved
Patient 37	Improved	Improved	Improved	Improved	Improved
Patient 38	Improved	Improved	Improved	Improved	Improved
Patient 39	Improved	Improved	Improved	Improved	Improved
Patient 40	Improved	Improved	Improved	Improved	Improved
Patient 41	Improved	Improved	Improved	Improved	Improved
Patient 42	Improved	Improved	Improved	Improved	Improved
Patient 43	Improved	Improved	Improved	Improved	Improved
Patient 44	Improved	Improved	Improved	Improved	Improved
Patient 45	Improved	Improved	Improved	Improved	Improved
Patient 46	Improved	Improved	Improved	Improved	Improved
Patient 47	Improved	Improved	Improved	Improved	Improved
Patient 48	Improved	Improved	Improved	Improved	Improved
Patient 49	Improved	Improved	Improved	Improved	Improved
Patient 50	Improved	Improved	Improved	Improved	Improved
Patient 51	Improved	Improved	Improved	Improved	Improved
Patient 52	Improved	Improved	Improved	Improved	Improved
Patient 53	Improved	Improved	Improved	Improved	Improved
Patient 54	Improved	Improved	Improved	Improved	Improved
Patient 55	Improved	Improved	Improved	Improved	Improved
Patient 56	Improved	Improved	Improved	Improved	Improved
Patient 57	Improved	Improved	Improved	Improved	Improved
Patient 58	Improved	Improved	Improved	Improved	Improved
Patient 59	Worsened	Improved	Improved	Improved	Improved
Patient 60	Improved	Improved	Improved	Improved	Improved
Patient 61	Improved	Improved	Improved	Improved	Improved
Patient 62	Improved	Improved	Improved	Improved	Improved
Patient 63	Improved	Improved	Improved	Improved	Improved
Patient 64	Improved	Improved	Improved	Improved	Improved
Patient 65	Improved	Improved	Improved	Improved	Improved
Patient 66	Improved	Improved	Improved	Improved	Improved
Patient 67	Improved	Improved	Improved	Improved	Improved
Patient 68	Improved	Improved	Improved	Improved	Improved
Patient 69	Improved	Improved	Improved	Improved	Improved
Patient 70	Improved	Improved	Improved	Improved	Improved
Patient 71	Improved	Improved	Improved	Improved	Improved
Patient 72	Improved	Improved	Improved	Improved	Improved
Patient 73	Improved	Improved	Improved	Improved	Improved
Patient 74	Improved	Improved	Improved	Improved	Improved
Patient 75	Improved	Improved	Improved	Improved	Improved
Patient 76	Improved	Improved	Improved	Improved	Improved
Patient 77	Improved	Improved	Improved	Improved	Improved
Patient 78	Improved	Improved	Improved	Improved	Improved
Patient 79	Improved	Improved	Improved	Improved	Improved
Patient 80	Improved	Improved	Improved	Improved	Improved

Pictures of these patients' posed smiles, both before and after therapy, were taken with their heads held in their usual positions. The photos were imported into photo editing software (Adobe Photoshop, version 7, Adobe Frameworks, San Jose, California), and made some adjustments with the built-in vertical (from the nose tip to the delicate tissue pogonion) and diagonal (from the zygomatic prominence) guides. A Visual Simple Scale and a Graph of Facial Stylish References both created in Adobe Photoshop were used to evaluate the photos.

The patients' smiles were initially assessed in Adobe Photoshop using a facial aesthetics reference diagram. Each tooth is encased in a frame that carefully follows its contours. DFAR, in its original name, alludes to the highest apical points of the gingival contour, known as apexes. The current reappraisal was performed to include the positions of the gingival papillae's papillary tips and highlight the contact places. When you connect these locations, you will get lines that may be used as benchmarks for grading how a smile is perceived. Referencing the idea of dental connections, the band formed by the papillary line's relationship to the line of the contact point is called the connector band. Six horizontal grin lines are created when the top and lower lip contours are used. In order from the neck down, they are as follows: (a) the cervical line; (b) the papillary line; (c) the contact points line; (d) the incisal line; (e) the upper lip line; and (f) the lower.

The second half of this research compared the perceived beauty of smiles before and after orthodontic treatment using a jury composed of five male orthodontists, five male general dentists, and five male laymen. A slide presentation created in Microsoft Office PowerPoint 2010 (Microsoft® Corp., Redmond, WA) and including the standardized pictures was used. All the photos had their identifying details blurred off, and then they were given random numbers between 1 and 140. Members of the panel were given a quick, illustrative overview of the Visual Analogue Scale. The judges used a 100mm Visual Analogue Scale on which they assigned points ranging from 0 (very ugly) to 10 (extremely attractive) for each smile they saw. It took the judges just 20 seconds only to rate each shot.

SPSS (Statistical Package for the Social Sciences for Windows, Version 16.0, Chicago, SPSS Inc.) was used to input and evaluate evaluations from the sample size. Each assessor group employed an independent t-test and a paired samples t-test for intergroup and intergroup comparisons. The threshold of statistical significance was set at P < 0.05.

## Results

For the purpose of this study, the digital archives of the Department of Orthodontics and Dentofacial Orthopaedics were searched for photos of posed smiles taken before and after orthodontic treatment on a total of 80 patients. The consonance of the grin arc, relative gingival zenith positions of the front teeth, and tooth length were the factors considered. Height of the incisal edges relative to the rest of the front teeth, width of the connector band in the front six teeth, and height of the front tooth's tips.

T-test for comparing means of pre-treatment assessments by different raters, independent samples general dentists' opinions varied from those of orthodontic specialists and laypeople, as shown by photographs. The P-values for these variations were less than 0.001 and less than 0.025, respectively, indicating their statistical significance. Table 2 shows that orthodontists, general dentists, and general person's opinions on the attractiveness of smiles before orthodontic treatment were similar where each parameter was compared within groups.

**Table 2:** Pre-treatment assessment comparison using independent samples t-test to compare mean values from different raters

Variables	N	Mean	Standard deviation	P-value
Orthodontist	80	3.11	0.89	<0.001
Dentist	80	3.23	0.76	
Orthodontist	80	3.56	0.88	0.345
General person	80	3.35	1.11	
Dentist	80	3.78	0.67	0.014
General person	80	3.39	1.45	

Test for mean differences amongst evaluators using independent samples t-statistics after treatment analysis of the photographs revealed discrepancies in the opinions of orthodontists, general dentists, and general persons. Table 3 shows that when comparing the evaluations of orthodontists and dentists, as well as orthodontists and general persons and dentists and general persons, there are statistically significant differences, with P-values of 0.001, 0.001, and 0.001, respectively in post-treatment assessment.

**Table 3:** Independent samples t-test to compare mean values between assessors in the post-treatment assessment comparison

Variables	N	Mean	Standard deviation	P-value
Orthodontist	80	6.23	0.34	<0.001
Dentist	80	5.09	0.56	
Orthodontist	80	6.24	0.89	0.00
Layman	80	5.56	0.45	
Dentist	80	5.06	0.76	0.01
Layman	80	5.57	0.34	

Comparative studies

The pre-treatment and post-treatment assessment improvement in each assessor group was compared using a t-test. Orthodontists, general dentists, and general persons all agreed that, when treatment was completed, the patient's smile was noticeably better than it had been before. Table 4 demonstrates that the enhancements in smile aesthetics were statistically significant across all three groups, with a P-value of 0.001.

**Table 4:** Paired samples t-test to compare the improvement in the pre-treatment and post-treatment assessment in each assessor group

Variables	Assessment	N	Mean	Standard deviation	P-value
Orthodontist	Post-treatment	80	6.23	0.98	<0.001
	Pre-treatment	80	3.34	0.67	
Dentist	Post-treatment	80	5.07	0.78	<0.001
	Pre-treatment	80	3.47	0.45	
General person	Post-treatment	80	5.45	0.67	<0.001
	Pre-treatment	80	3.39	1.11	

## Discussion

The primary goal of any cosmetic dentistry procedure is to improve the appearance of the patient's smile. Despite its significance, however, the underlying components of a grin are seldom examined. These traits are either fixed or malleable depending on the situation since they make up different elements of each person. So, dentists can only make judgments about these traits without any control over them. A person's beauty is more determined by their grin than by the relaxed state of their facial soft tissues. According to Proffit et al. [6], there are two primary types of smiles: the posed or social grin, and the emotional smile. The posed grin is exhibited to the public because it is the one most focused on during orthodontic diagnosis (the social smile) and because it is easy to replicate. Therefore, in this study, we used photographs of staged smiles to assess how much orthodontic therapy improved smile attractiveness. Full-face images have been used in studies by Shaw et al. [7], Mackley et al. [8], Moore et al. [9], and Hunt et al. [10]. The problem, as pointed out by Shaw et al. [2], is that the background facial attractiveness is often more assertive than the individual dental condition.

When trying to find a visual representation of an issue, people often make a number of assumptions and follow a set of rules that may lead to either an underestimating of faults or an overvaluation of rules, resulting in paradigms that are not based on hard scientific evidence. Using tried-and-true techniques may increase success odds and reduce or do away with mistakes in execution altogether. An additional diagnostic tool that works well for this is the DFAR. Seventy patients were selected at random from among those who had had orthodontic therapy for various forms of malocclusion. After patients were treated, they were photographed with a staged grin in front of a white background to compare results. Proffit et al. [6] defined the consonance of the grin arc as the angle formed between the incisal margins of the upper front teeth and the lower lip when the two are joined in a friendly smile. Sixty-six out of seventy evaluated cases showed an increase in grin arc consonance. For the other four, two examples showed no change and two indicated worsening consonance, presumably owing to incorrect bracket alignment. Those patients that did not improve despite treatment had excellent consonance, to begin with. However, two deterioration examples highlight the significance of precise section location, the must to routinely be auditing smile feel throughout the doing and itemizing phases of therapy, and the necessity of correcting defects in section arrangement. It was found that the incisal margins of most patients grew in height during the course of the examination, demonstrating the ease with which this border may be measured while the patient is held. McLaughlin et al. [11] recommend setting the sections for the central incisor and canine at the same distance from the incisal edge, with the section for the lateral incisor located 0.5mm incisal to the central incisor. It was determined where the gingival emphasis of the upper and lower front teeth should be by referencing the gingival line on the Facial Stylish Reference Graph. The gingival line is formed when the gingival peaks of the canines, maxillary laterals, and central incisors come together.

Sixty-four of the cases treated showed an increase to a convex position of the gingival zenith, whereas two instances deteriorated and four cases showed no change. Since a concave gingival line is less aesthetically pleasing, further operations like gingivoplasty and selective grinding may be performed to boost the attractiveness of a patient's smile. Contact lines were used to assess the connection band. However, the contact areas between the teeth are not shown by these lines. The connector band tightened after therapy in all of the instances examined in this research. While precise measurements were out of the question because of the inherent magnification inaccuracy of the pictures, the connector's visual "Hand Glider" form was used as a proxy for quality. A vertical line was drawn from the apparent midpoint of the incisal edges to the apparent midpoint of the gingival zenith to find the relative apex of the teeth. The value of this parameter was calculated by observing the angle at which the vertical lines began to deviate from the face's midline. When applied to real-world data, this statistic showed a general upward trend.

The second section of this research project assessed the smile's attractiveness. The perioral pictures were judged on a scale from 1 to 10. Parekh et al. [12] study discovered that male and female orthodontists judged smile aesthetics differently. Because of the potential for bias between male and female reviewers, we selected an all-male review panel; nevertheless, comparing the ratings of male and female reviewers is beyond the scope of this particular investigation. The mean scores among assessor groups were compared for the post-treatment photos using an independent samples t-test (Table 1). None of the three sets of raters in this research agreed with one another, whether they were orthodontists, general dentists, or the general public.

According to Flores et al. [13], Aesthetic appreciation differs from person to person since it is shaped by their unique life history and network of relationships. Different perspectives on what constitutes physical attractiveness exist between the general public and the medical and cosmetic industries for similar reasons. Annemieke et al. [14] showed that orthodontists and their patients had different assessments of the same smiles. Kokich et al. [15] and Roden-Johnson et al. [16] concur with the study's findings that orthodontic specialists, ordinary dentists, and the general public have different aesthetic preferences. Before and after photos of a patient's smile were compared using the paired samples t-test to see how much of an improvement there was in smile aesthetics (Table 2). After receiving therapy, participants' smiles were shown to have improved significantly in this research. The levels of satisfaction indicated by orthodontists (P=0.001), general dentists (P=0.001), and the general public (P=0.001) all increased significantly.

We observed that the beauty of a smile may be significantly enhanced with fixed orthodontic treatment and that the assessment of a smile's attractiveness varies across orthodontists, general dentists, and the general public. Some factors, such as the consonance of the smile arc and the relative height of the incisal margins, might worsen with time, as shown by objective smile aesthetics evaluation. Orthodontists, in their capacity as smile designers, need to be aware that fixed orthodontic treatment may have a major impact on the aesthetics of patients' smiles [17,18]. At the end of orthodontic treatment, when the finer details are being worked out, the doctor should take into account how the patient's smile looks [19]. More research is needed to determine if orthodontic treatment methods (extraction vs. non-extraction, intrusion vs. retraction, etc.) have an effect on smile aesthetics.

Simply fixing individual teeth is no longer acceptable in the profession of dentistry nowadays. A greater number of patients are requesting final results that are not just mechanically and physiologically sound but also aesthetically beautiful. Bleaching, bonding, and veneering have made it possible to undergo a wide range of cosmetic dental procedures in addition to healing and reconstructing the damaged dentition, frequently reversing the visible signs of aging. Considering these variables, a study by Calamia et al. employed the usage of a Comprehensive Esthetic Evaluation Form for smile design and treatment planning [20]. Although a very different methodology than what we employed for our study, the above study did observe very good patient compliance which warrants further research using the methodology.

Orthodontists are increasingly utilizing the rapidly-growing levels of technology, with smile designing and aesthetics being at the forefront of this treatment approach. A systematic review and meta-analysis regarding this modality were conducted by Ahmed et al. [21], where the study aimed to assess the efficiency of dental practices' use of digital smile design approaches. These methods were employed in various branches of medicine, and the clinicians examined and categorized each of those branches as well as assessed the dependability and predictability of these digital methods. The utilization of technology (in the form of usage of cameras) in this study was on a similar line as ours, although the rest of the study design differed quite a lot from ours.

A qualitative research approach with respect to establishing the importance of aesthetics in the field of orthodontic treatment might also be viable along with all of the different methodologies that we have discussed since a qualitative approach makes it possible to comprehend the meaning of smiles and their significance in our daily encounters. Qualitative approaches focus on gathering experiences and articulated meanings through conversations, open-ended inquiries, and interviews [22,23]. Teenagers with malocclusion were particularly ashamed to expose their teeth when speaking to others, according to one prominent example [24]. Not all teenagers, though, have sensitive teeth when they smile [25]. Additionally, despite the fact that it may appear like an easy task, qualitative interviews gave insight into the anxiety experienced by orthodontic patients when asked to smile for a photograph [26]. According to a study, these patients' grin expressions may not only be related to their pre-treatment worries but also to the presence of unfavorable white spots lesions [27].

Since qualitative data is abundant, there are numerous strategies for obtaining it from online platforms and social media. After all, smiling might be viewed as a way to spread optimism on social media and even as a way to speed up the hiring process on those sites [28]. Whether someone chooses to smile with their teeth visible or not, there are a variety of ways to do so on social media, as has been noted in earlier study results [29,30]. For instance, extracting tweets showed that certain dentofacial traits might be crucial in changing smiling behavior to prevent bullying [31]. Additionally, people with malocclusion voiced worries about their pictures appearing on social networking sites [32]. Social media may distract users, preventing them from making expressive facial expressions like smiles [33].

In orthodontic research, questionnaires are another common instrument that is used widely [34]. Incorporating specialized or pre-designed questionnaires into smile studies could provide an effective and uncomplicated way to gauge how patients, doctors, and laypeople perceive smiles. For instance, the smile esthetics-related quality of life questionnaire [35] examines the sub-domains of confidence with one's smile, awareness of altered smile aesthetics, and the impact of smile aesthetics in a social context. A rapid tool for such investigations, the five-item Smile Aesthetics Pleasure Scale (SASS) questionnaire, can be used to quantify smile satisfaction in adults [36]. For orthodontic patients, having a beautiful smile is crucial, and many important factors are frequently thought to have an impact on overall satisfaction [37]. Many times, elements influencing happiness with smiles are analyzed, categorized, and differentiated using questionnaire-based surveys and visual analog ratings. In one study, participants responded to a specific questionnaire about the appeal of their smiles by gazing into a picture of their natural grins. The findings showed that individuals were happy when their smiles displayed all of their teeth and some gingiva, but they were unhappy when their smiles displayed an unbalanced amount of gingiva [38]. According to surveys on how buccal corridors are perceived during smiles, orthodontists and prosthodontists greatly value smiles with them, in contrast to laypeople who think smiles with and without them are similar [39]. One intriguing study found that orthodontic specialists were more receptive to gummy smiles than dentistry students [40]. Other research looking at the aesthetics of smiles with displayable recession revealed that when dental professionals scored the smiles on a visual analog scale, they were aware of these aesthetic restrictions (i.e., the recession's presence) [41]. The Dental Aesthetic Index (DAI) and the subjective aesthetic smile presentation were successfully linked by questionnaire-based investigations [42]. According to one study, when utilizing the Psychosocial Impact of Dental Aesthetics Questionnaire to evaluate the psychosocial impact of smile aesthetics, the severity of malocclusion is a key predictor [43].

The study limitations involve the use of a limited sample size and a particular area of the population. The study has not considered various other parameters of esthetics such as smile line and extraction or non-extraction cases into considerations so can be included in further research.

## Conclusions

Orthodontic therapy improved several facets of a smile that contribute to its aesthetic appeal. These included the level of the incisal edges of the front teeth in respect to one another, the consonance of the grin bend, the overall tip of the teeth, the placement of the gingival apex in relation to one another, and the breadth of the connector band. The aesthetic value of patients' smiles was significantly improved by orthodontic treatment, according to orthodontists, general dentists, and average people. Clinicians can use the facial aesthetic reference diagram to objectively assess the elements influencing a patient's smile and carry out the necessary therapies. The findings of this study lend credence to the notion that evaluation of the smile's aesthetics should occur during the latter phases of orthodontic treatment when minor changes are being made.
